# Acute tau knockdown in the hippocampus of adult mice causes learning and memory deficits

**DOI:** 10.1111/acel.12775

**Published:** 2018-05-10

**Authors:** Ramon Velazquez, Eric Ferreira, An Tran, Emily C. Turner, Ramona Belfiore, Caterina Branca, Salvatore Oddo

**Affiliations:** ^1^ Arizona State University‐Banner Neurodegenerative Disease Research Center at the Biodesign Institute Arizona State University Tempe AZ USA; ^2^ School of Life Sciences Arizona State University Tempe AZ USA

**Keywords:** AD, Alzheimer's disease, BDNF, NFTs, spine density, Tangles

## Abstract

Misfolded and hyperphosphorylated tau accumulates in several neurodegenerative disorders including Alzheimer's disease, frontotemporal dementia with Parkinsonism, corticobasal degeneration, progressive supranuclear palsy, Down syndrome, and Pick's disease. Tau is a microtubule‐binding protein, and its role in microtubule stabilization is well defined. In contrast, while growing evidence suggests that tau is also involved in synaptic physiology, a complete assessment of tau function in the adult brain has been hampered by robust developmental compensation of other microtubule‐binding proteins in tau knockout mice. To circumvent these developmental compensations and assess the role of tau in the adult brain, we generated an adeno‐associated virus (AAV) expressing a doxycycline‐inducible short‐hairpin (Sh) RNA targeted to tau, herein referred to as AAV‐ShRNATau. We performed bilateral stereotaxic injections in 7‐month‐old C57Bl6/SJL wild‐type mice with either the AAV‐ShRNATau or a control AAV. We found that acute knockdown of tau in the adult hippocampus significantly impaired motor coordination and spatial memory. Blocking the expression of the AAV‐ShRNATau, thereby allowing tau levels to return to control levels, restored motor coordination and spatial memory. Mechanistically, the reduced tau levels were associated with lower BDNF levels, reduced levels of synaptic proteins associated with learning, and decreased spine density. We provide compelling evidence that tau is necessary for motor and cognitive function in the adult brain, thereby firmly supporting that tau loss‐of‐function may contribute to the clinical manifestations of many tauopathies. These findings have profound clinical implications given that anti‐tau therapies are in clinical trials for Alzheimer's disease.

## 1 INTRODUCTION

The microtubule‐associated protein tau is linked to several neurodegenerative disorders. Under normal physiological conditions, tau promotes microtubule assembly and stabilization (Weingarten, Lockwood, Hwo & Kirschner, 1975). Through its interaction with microtubules, tau plays a crucial role in the regulation of axonal transport, which is critical for neuronal function (Ashe, 2007). Tau also maintains the dynamic cytoskeleton required for adult hippocampal neurogenesis and promotes axonal outgrowth (Fuster‐Matanzo, Llorens‐Martín, Jurado‐Arjona, Avila & Hernández, 2012). Recently, there has been growing appreciation for other roles of tau (Ashe, 2007). For example, tau can directly interact with the plasma membrane and with several tyrosine kinases, thereby regulating selective intracellular pathways (Fulga et al., 2007). Additionally, intense synaptic activity drives tau into dendritic spines, suggesting that tau may participate in spine remodeling that underlies synaptic plasticity (Frandemiche et al., 2014).

Pathological tau, by contrast, exhibits altered solubility properties, forms filamentous structures, and is abnormally phosphorylated (Khan & Bloom, 2016). Hyperphosphorylated tau accumulates to form neurofibrillary tangles (NFTs), which are hallmark lesions of several neurodegenerative disorders including Alzheimer's disease (AD), frontotemporal dementia with Parkinsonism linked to chromosome 17, Pick's disease, progressive supranuclear palsy, and corticobasal degeneration (Khan & Bloom, 2016). Collectively, these disorders are known as tauopathies. The prevalent hypothesis in tauopathies has been that the misfolding and hyperphosphorylation of tau causes its accumulation, thereby producing cognitive deficits. For example, recent studies have shown that somatodendritic accumulation of tau in AD may impair cognitive function (Li & Götz, 2017). However, hyperphosphorylated tau dissociates from microtubules, compromising their stability and function (Brunden, Trojanowski, Smith, Lee & Ballatore, 2014). To this end, hyperphosphorylation of tau impairs axonal transport of organelles, which may result in synapse starvation, depletion of ATP, and neuronal damage (Reddy, 2011). Overall, there is an ongoing debate in the literature as to whether tau‐mediated behavioral deficits are due to tau toxic gain‐of‐function or to tau loss‐of‐function (Trojanowski & Lee, 2005). Data from animal models suggest that both mechanisms may be involved. For example, early tau transgenic mice indicated that the behavioral deficits correlated with the degree of NFTs (Trojanowski & Lee, 2005). In contrast, inducible tau transgenic mice showed that NFTs are not sufficient to cause behavioral deficits (SantaCruz et al., 2005), suggesting that tau loss‐of‐function may account for cognitive deficits.

While a wealth of data is available on the role of tau in the formation of toxic inclusions, less is known about its physiological function in the adult brain. Four independent lines of full tau knockout mice have been generated prior to this study, and no significant behavioral or neuropathological phenotype has been reported (Dawson et al., 2010; Fujio et al., 2007; Harada et al., 1994; Ke et al., 2012; Tucker, Meyer & Barde, 2001). The relatively mild phenotype is likely due to compensatory events during development and to the redundancy associated with different microtubule‐binding proteins (Denk & Wade‐Martins, 2009; Ke et al., 2012). To bypass possible compensations that may arise from knocking out tau during development and evaluate the role of tau in the adult brain, we generated an adeno‐associated virus expressing a doxycycline‐inducible short‐hairpin RNA targeted to tau (AAV‐ShRNATau). We performed bilateral stereotaxic injections of the virus into the hippocampus of adult mice. Our approach circumvents the developmental compensation issues observed in tau knockout models and shows that reducing tau levels during adulthood impairs both cognitive and noncognitive behavior.

## 2 RESULTS

### 2.1 Acute tau knockdown impairs motor coordination, endurance, and spatial memory

**Figure 1:**
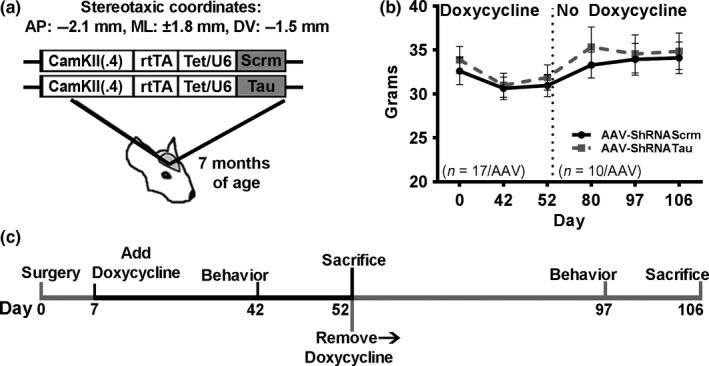
Tau knockdown does not alter body weight. (a) Depiction of the two adeno‐associated viruses (AAVs) that were bilaterally injected into CA1 of the hippocampus of 7‐month‐old C57Bl6/SJL mice. (b) There was no significant difference in body weight between the two AAV groups throughout the first 52 days of the experiment when all mice were on doxycycline. Additionally, there was no significant difference in body weight between the two AAV groups on Days 53 and 106, when all remaining mice were off doxycycline. (c) Diagram of the experimental design. Gray bars on the diagram indicate that the expression of the ShRNAs was turned off. Thirty‐four mice underwent bilateral stereotaxic surgeries, and half were injected with the AAV‐ShRNATau (*n* = 17), while the other half were injected with the control virus (AAV‐ShRNAScrm; *n* = 17). Seven days later, all animals were put on doxycycline to initiate the expression of the ShRNATau and the ShRNAScrm. All animals were behaviorally tested 35 days post doxycycline, and seven mice/group were sacrificed at the end of testing, on Day 52. The remaining mice (*n* = 10/group) were kept alive and taken off doxycycline for 45 days to turn off expression of the two ShRNAs. Behavioral testing lasted 9 days, so these animals were off doxycycline for a total of 53 days

To reduce tau expression in adult brains, we generated an adeno‐associated virus (AAV) expressing a doxycycline‐inducible short‐hairpin (Sh) RNA targeted to tau. The AAV was generated with the following construct: AAV1‐CamKII‐rtTA‐Tet/U6‐Tau‐ShRNA. The CamKII promoter allows for neuron‐specific expression of the ShRNA. We also generated a virus expressing a scramble ShRNA sequence, AAV1‐CamKII‐rtTA‐Tet/U6‐Scrm‐ShRNA (Figure 1a). The final viral titer was 3.8 × 10^13^ GC/ml. We injected 2.0 μl/hemisphere of the AAVs into CA1 of the hippocampus of 7‐month‐old C57Bl6/SJL mice (*n* = 17/group; Figure 1a). We assessed the mice body weight at Day 0 (before the surgeries), Day 42, and Day 52, and found no significant effect (*F*_(1,64)_ = 1.025, *p *>* *.05; Figure 1b). As detailed below, we sacrificed seven mice per group at Day 52 and assessed body weight of the remaining mice at Days 80, 97, and 106 and found no significant differences (*F*_(1,34)_ = 1.491, *p *>* *.05; Figure 1b–c). These results show that neither surgery nor doxycycline affected body weight throughout the study.

**Figure 2:**
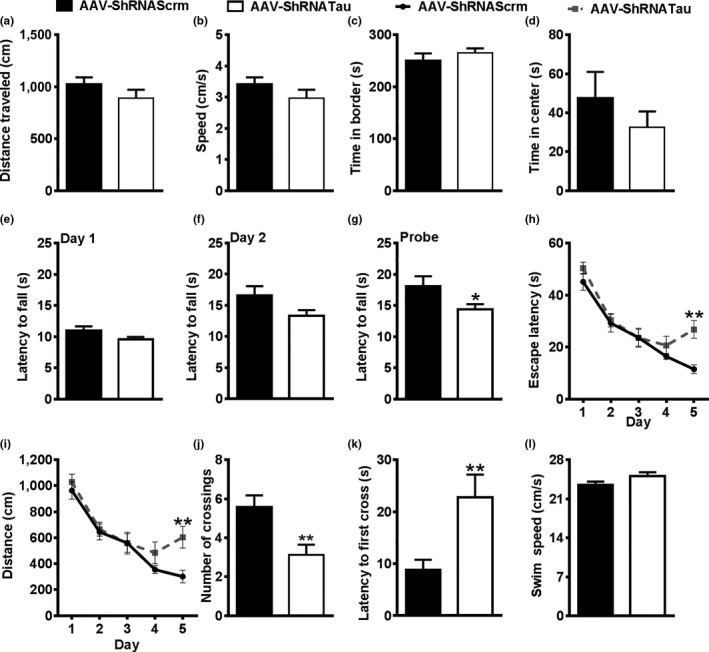
Acute tau knockdown impairs motor coordination, endurance, and spatial memory. (a–b) On day 42, all mice were placed in an open‐field cage for 10 min to measure mobility and anxiety‐like activity. No significant difference in distance traveled and speed (cm/s) was detected between the two AAV groups. (c–d) No significant difference in the time spent in the periphery or the center of the open‐field cage was detected between the two AAV groups. (e–f) The mice were next tested in the rotarod task to assess motor coordination and endurance. On Days 1 and 2, the rod spins at a rate of 0.75 rpm/s for the first 20 s and then remains at 15 rpm for the remaining 70 s (90 s total). No differences were detected between the two AAV groups on latency to fall from the spinning rod on Day 1 or 2. (g) On Day 3, when the rod accelerates at 1 rpm/s (max 90 s), AAV‐ShRNATau mice stayed on the spinning rod significantly less than the AAV‐ShRNAScrm group (*p *<* *.05). (h–l) The mice were then tested on the Morris water maze (MWM) task to assess spatial reference learning and memory. AAV‐ShRNATau mice are significantly impaired compared to AAV‐ShRNAScrm mice on Day 5 as evident by an increase in latency to the platform (*p *<* *.01) and distance traveled (*p *<* *.01). On Day 6, the platform was removed and animals were given a 60‐s probe trial to explore the pool. AAV‐ShRNATau mice crossed the platform location significantly less (*p *<* *.01) and took longer to cross the platform location than the AAV‐ShRNAScrm mice (*p *<* *.01), illustrating spatial memory deficits. No significant difference in swim speed (cm/s) was detected between the two AAV groups. (Data are means ± *SE*. *p <* *.05; **p <* *.01)

To induce the expression of the AAVs, we gave mice 2 mg/ml doxycycline in their drinking water, 7 days after surgeries. To determine the effects of acute tau knockdown on behavioral performance, we tested the mice in a series of cognitive and noncognitive behavioral tests 35 days after doxycycline administration (Figure 1c). We used the open‐field activity test to measure general motor function and anxiety‐like behavior. We found that spontaneous activity and gross motor function were similar between the two groups, as indicated by both an equal distance traveled (*t*_(32)_ = 1.721, *p *>* *.05; Figure 2a) and average speed (*t*_(32)_ = 1.704, *p *>* *.05; Figure 2b) in the activity chamber. To evaluate general anxiety and stress, we measured thigmotaxis and the time spent in the center of the activity chamber. We found that the time spent in the periphery of the activity chamber (*t*_(32)_ = 0.826, *p *>* *.05; Figure 2c) and the center (*t*_(32)_ = 0.924, *p *>* *.05; Figure 2d) was similar between the two groups. These data indicate that the induction of the AAV‐ShRNATau does not affect general motor function or anxiety‐like behavior.

To assess motor coordination and endurance, we trained mice on the rotarod task for three consecutive days. During the first two training days, the rod spins at a rate of 0.75 rpm/s for the first 20 s and then remains constant at 15 rpm for the remaining 70 s. We found that the latency to fall was not statistically different between the groups on Day 1 (*t*_(32)_ = 3.164, *p *>* *.05; Figure 2e) or Day 2 of the task (*t*_(32)_ = 3.678, *p *>* *.05; Figure 2f). On Day 3, we performed the probe trials by giving mice six consecutive trials during which the rod accelerated at 1 rpm/s for up to 90 s. We found that AAV‐ShRNATau mice had a significantly lower latency to fall compared to AAV‐ShRNAScrm mice (*t*_(32)_ = 4.243; *p *<* *.05; Figure 2g). These results indicate that 35 days of tau knockdown is sufficient to impair motor coordination and endurance.

To determine the effect of acute tau knockdown on spatial reference learning and memory, we tested all mice on the Morris water maze (MWM) for 6 consecutive days. During the first 5 days, mice received four trials per day to locate a hidden platform using extra‐maze cues. We found a significant group by latency interaction (*F*_(1,128)_ = 2.658, *p *<* *.05; Figure 2h) and a group by distance traveled interaction (*F*_(1,128)_ = 2.499, *p *<* *.05; Figure 2i). Post hoc analysis using Bonferroni correction indicates that the AAV‐ShRNATau mice were significantly impaired on Day 5 of the learning trial as evident by both an increased latency to the platform (*p *<* *.01) and distance traveled to the platform location (*p *<* *.01). Even though it may seem unusual that a group of mice is statistically different from another only during the last day of training, other groups have reported similar findings (e.g., Darcet et al., 2014; Pittenger et al., 2002; Sun et al., 2014). On Day 6, we removed the platform and tested the mice in a 60‐s probe trial, to assess spatial memory. We found that the AAV‐ShRNATau mice crossed the platform location significantly fewer times than the AAV‐ShRNAScrm mice (*t*_(32)_ = 8.856; *p *<* *.01; Figure 2j) and had a significantly higher latency to first cross the platform location (*t*_(32)_ = 7.920; *p *<* *.01; Figure 2k), illustrating spatial memory deficits. Notably, we analyzed swim speed during the probe trials and found no significant differences between the two groups (*t*_(32)_ = 1.901; *p *>* *.05; Figure 2l). Together, these data indicate that AAV‐ShRNATau mice can swim as well as AAV‐ShRNAScrm mice while having a motor deficit in the rotarod. To this end, different muscles and movements are needed to perform these two different tasks; thus, this dissociation is not surprising (Deacon, 2013; Vorhees & Williams, 2006). After behavioral testing, we sacrificed seven mice/group and extracted their hippocampi for further analyses. We removed doxycycline from the drinking water of the remaining 10 mice/group, to halt the expression of the ShRNAs, and let the mice age for an additional 45 days, after which we reassessed mice behaviorally (Figure 1c).

**Figure 3:**
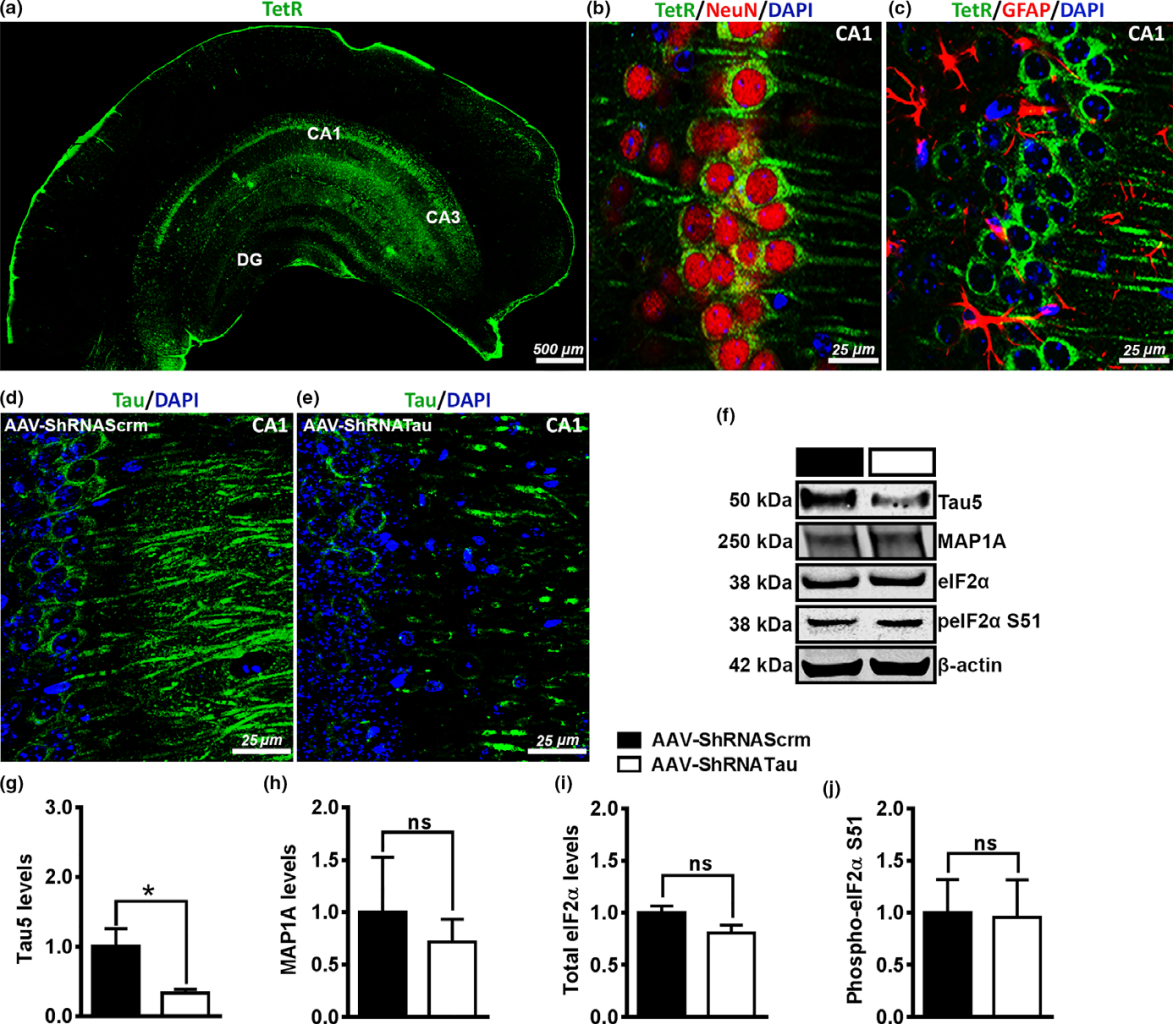
Acute tau knockdown did not lead to upregulation of other microtubule‐associated proteins. We verified the expression of the AAVs by immunostaining sections of mice with a tetracycline repressor protein (TetR) antibody; TetR binds to the essential component of the Tet‐On transactivator protein of the AAVs. (a) Photomicrograph of a coronal section from an AAV‐ShRNATau‐injected mouse shows infection predominately in the CA1 region with some leakage into the cortex. (b) Double labeling for TetR and neuron‐specific marker NeuN with nuclear marker DAPI shows co‐expression of TetR in neurons. (c) Double labeling for TetR and GFAP with DAPI shows no co‐expression of the AAVs with glial cells. (d‐e) Tau labeling shows reduced expression in CA1 in the AAV‐ShRNATau mice compared to the AAV‐ShRNAScrm mice. (f) Representative Western blots of proteins extracted from the hippocampi of mice injected with the tau or scrambled ShRNA and probed with the indicated antibodies. (g) Quantitative analyses of the blots indicated a 68 ± 16% reduction in tau levels after expression of the AAV‐ShRNATau compared to control mice (*p *<* *.05). (h) There was no significant difference in MAP1A levels between the AAV‐ShRNATau and Scrm mice. (i‐j) There was no significant difference in both total eIF2α levels and phosphorylated (p) eIF2α at S51 levels between the AAV‐ShRNATau and Scrm mice. (Data are means ± *SE*. *p <* *.05, ns non significant)

To assess the viral diffusion, we immunostained sections of AAV‐ShRNATau and ShRNAScrm mice with an antibody against the tetracycline repressor protein (TetR). We found TetR immunoreactivity predominantly in the CA1 region of the hippocampus and, to a lesser extent, in the surrounding cortex (Figure 3a). To determine the cell type infected by the AAVs, we co‐labeled hippocampal sections with TetR and the neuron‐specific marker, NeuN, or the astrocyte marker, GFAP. The majority of the virus was expressed in neurons as indicated by the co‐localization between TetR and NeuN and the lack of co‐localization between TetR and GFAP (Figure 3b–c). This finding is not surprising given that the TetR is under the control of the neuron‐specific CamKII promoter.

To determine whether the AAV‐ShRNATau reduces tau levels, we immunostained sections from both groups with an antibody against tau. We found a reduction in tau expression in the CA1 of AAV‐ShRNATau‐injected mice compared to the Scrm mice (Figure 3d–e). To better quantify these changes, we measured tau levels by Western blot and found a 68 ± 16% significant decrease in the steady‐state levels of tau in mice injected with the AAV‐ShRNATau compared to the mice injected with the Scrm AAV (*t*_(12)_ = 8.817, *p *<* *.05; Figure 3f–g). To determine whether the AAV‐ShRNATau induces any compensatory mechanisms upregulating other microtubule‐associated protein (MAPs), we measured MAP1A levels in the AAV‐ShRNAScrm and AAV‐ShRNATau mice that were on doxycycline. We found that acute tau knockdown did not lead to changes in MAP1A (*t*_(12)_ = 0.246, *p *>* *.05; Figure 3f,h). This is notable given that upregulation of MAP1A has been reported in many tau knockout mice (Denk & Wade‐Martins, 2009; Ke et al., 2012).

To determine whether there were nonspecific effects of the ShRNA that may have altered protein synthesis, we measured the levels of the eukaryotic initiation factor 2α (eIF2α) and its phosphorylation at S51 by Western blot. We focused on this initiation factor because when tissues are exposed to various forms of stress, there is an increase in phosphorylation of eIF2α at S51, which results in a general inhibition of protein synthesis (Holcik & Sonenberg, 2005). We found no significant differences in both total eIF2α (*t*_(12)_ = 3.847, *p *>* *.05) and phosphorylated eIF2α at S51 (*t*_(12)_ = 0.234, *p *>* *.05) between the AAV‐ShRNATau and AAV‐ShRNAScrm (Figure 3f,i–j), which suggests that the AAV‐ShRNATau did not reduce overall protein synthesis via activation of eIF2α.

### 2.2 Halting the expression of the AAV‐ShRNATau rescues behavioral deficits

**Figure 4:**
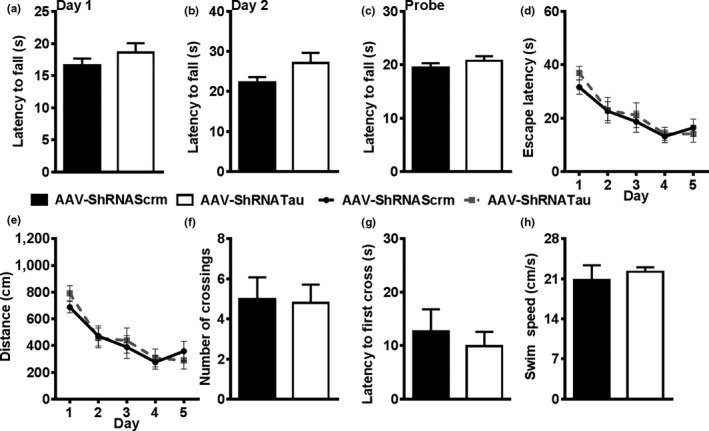
Halting the expression of the AAV‐ShRNATau rescues behavioral deficits. (a–c) Mice were retested on the rotarod task to assess motor coordination after removal of doxycycline. No significant differences were detected on the rotarod task on Day 1, Day 2, or the probe trial between the two AAV groups. (d–h) Mice were retested on the Morris water maze (MWM) task. We found no significant difference in latency to the platform and distance traveled between the two groups. On the probe trial, both AAV groups show an equal number of platform crosses and latency to cross the platform. No significant difference in swim speed was detected between the AAV groups for swim speed (cm/s)

To determine the effects of restoring tau levels on cognitive and noncognitive behavior, we assessed behavioral performance in the 10 mice in which we restored tau expression by turning off the expression of the AAV‐ShRNATau (Figure 1c). In the rotarod task, we found that both groups stayed on the spinning rod an equal amount of time during the training days (Day 1: *t*_(18)_ = 0.521, *p *>* *.05; Day 2: *t*_(18)_ = 0.854, *p *>* *.05; Figure 4a,b) and on the probe trial Day 3 (*t*_(18)_ = 0.266, *p *>* *.05; Figure 4c). These results illustrate a rescue in motor coordination and endurance 45 days after halting the expression of the AAV‐ShRNATau.

We also retested mice in the MWM. To avoid any retest effects (Vorhees & Williams, 2006), we switched the location of the escape platform to a different quadrant and replaced the extra‐maze cues. Both groups performed similarly in the 5 days of learning as evident by equal latency (*F*_(1,68)_ = 0.370, *p *>* *.05; Figure 4d) and distance traveled (*F*_(1,68)_ = 0.516, *p *>* *.05; Figure 4e) to find the escape platform. In the probe trial, we found no significant differences in platform crossings (*t*_(18)_ = 0.020, *p *>* *.05; Figure 4f), latency to first cross the platform location (*t*_(18)_ = 0.325, *p *>* *.05; Figure 4g), and swim speed (*t*_(18)_ = 0.306; *p *>* *.05; Figure 4h). These results show that resuming tau production restored spatial learning and memory in mice who had shown previous tau knockdown‐induced behavioral deficits. Collectively, our results highlight the important role of tau and suggest that tau loss‐of‐function may contribute to the cognitive and noncognitive deficits observed in various tauopathies.

### 2.3 Acute tau knockdown leads to synaptic deficits

**Figure 5:**
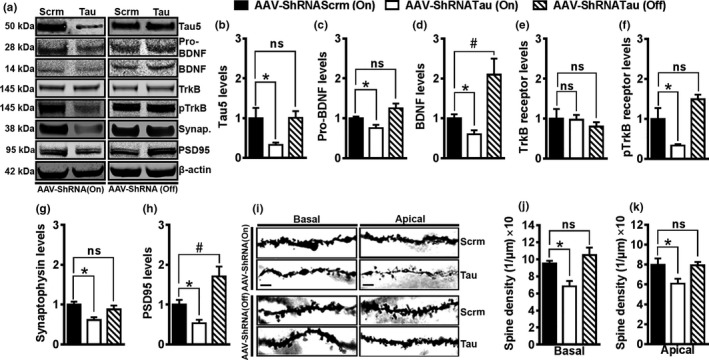
Acute tau knockdown reduces synaptic makers and dendritic spine density. (a) Representative immunoblot of proteins extracted from the hippocampi of mice injected with the tau or scrambled ShRNA and probed with the indicated antibodies. (b) Quantitative analyses of the blots indicated a 68 ± 16% reduction in tau levels after expression of the AAV‐ShRNATau compared to control mice (*p *<* *.0167). Turning off the expression of the AAV‐ShRNATau returned tau levels to baseline. (c) There was a 25 ± 13% significant reduction in the levels of immature pro‐BDNF after expression of the AAV‐ShRNATau compared to control mice (*p *<* *.0167). Turning off the expression of the AAV‐ShRNATau returned pro‐BDNF levels to baseline. (d) There was a 40 ± 17% significant reduction in the levels of mature BDNF after expression of the AAV‐ShRNATau compared to control mice (*p *<* *.0167). Turning off the expression of the AAV‐ShRNATau increased the levels of BDNF by 103 ± 32% compared to control mice (*p *<* *.0167), indicating an increase over baseline. (e) There was no significant difference in the total level of TrkB receptors among the three groups. (f) There was a 67 ± 27% significant reduction in phosphorylated TrkB receptor at Tyr816 levels after expression of the AAV‐ShRNATau compared to control mice (*p *<* *.0167). Blocking the expression of the AAV‐ShRNATau returned pTrkB levels back to baseline. (g) There was a 41 ± 21% significant reduction in synaptophysin levels after expression of the AAV‐ShRNATau compared to control mice (*p *<* *.0167). Blocking the expression of the AAV‐ShRNATau returned synaptophysin levels to baseline. (h) There was a 46 ± 13% significant reduction in PSD95 levels after expression of the AAV‐ShRNATau compared to control mice (*p *<* *.0167). Blocking the expression of the AAV‐ShRNATau increased the levels of PSD95 by 70 ± 25% compared to control mice (*p *<* *.0167), indicating an increase over baseline. (i) Photomicrographs of basal and apical dendrites taken at 100× power. (j) There was a 28 ± 10% significant reduction in the basal dendrites spine density after expression of the AAV‐ShRNATau compared to control mice (*p *<* *.0167). Blocking the expression of the AAV‐ShRNATau returned basal dendritic spine densities to baseline. (k) The spine density of apical dendrites was significantly reduced by 24 ± 14% after expression of the AAV‐ShRNATau compared to control mice (*p *<* *.0167). Blocking the expression of the AAV‐ShRNATau returned apical dendritic spine densities to baseline. Scale bar = 5 μm. Data are means ± *SE*. * indicates a significant change from the AAV‐ShRNATau (ON) group (*p *<* *.0167); # indicates a significant increase over baseline

After behavioral testing, we sacrificed the mice and removed their hippocampi for further analyses. These mice were analyzed together with the first cohort of mice sacrificed immediately after the first round of behavioral tests (Figure 1c). We measured tau levels in hippocampal homogenates and found a significant effect of group (*F*_(2,18)_ = 5.881, *p *<* *.0167; Figure 5a,b). Specifically, we found a 68 ± 16% reduction in tau levels after expression of the AAV‐ShRNATau. After blocking the expression of the AAV‐ShRNATau, tau levels returned to baseline. These results confirm that expression of the AAV‐ShRNATau reduces endogenous tau levels, which can be restored to baseline levels by blocking the expression of the AAV‐ShRNATau.

To understand the mechanism by which tau knockdown impairs learning and memory, we examined changes in various proteins known to be involved in memory formation and synaptic plasticity (Hu et al., 2011; Kowiański et al., 2017). We first examined the levels of hippocampal brain‐derived neurotrophic factor (BDNF) by Western blot. We found that the levels of pro‐BDNF were significantly different among the three groups (*F*_(2,18)_ = 8.671, *p *<* *.05; Figure 5a,c). Specifically, we found a 25 ± 13% reduction in pro‐BDNF levels after expression of the AAV‐ShRNATau compared to the control mice. After blocking the expression of the AAV‐ShRNATau, pro‐BDNF levels returned to baseline. Next we examined the hippocampal levels of mature BDNF and found a significant effect of group (*F*_(2,18)_ = 11.658, *p *<* *.05; Figure 5a,d). Specifically, we found a 40 ± 17% reduction in BDNF levels after expression of the AAV‐ShRNATau. Interestingly, we found that after restoring tau expression, BDNF levels were 103 ± 32% higher compared to control mice (*p *<* *.0167). BDNF is a secreted growth factor that binds to tropomyosin receptor kinase B (TrkB) receptor to initiate trophic retrograde signaling, which is critical for neuronal survival and synaptic plasticity (Kowiański et al., 2017). To determine whether the changes in BDNF levels would be reflected in the phosphorylation of its TrkB receptor, we measured total and phosphorylated levels of TrkB receptors by Western blot. While total TrkB levels were similar among the three groups (*F*_(2,18)_ = 0.411, *p *>* *.05), the levels of TrkB phosphorylated at Tyr816 were significantly different (*F*_(2,18)_ = 11.722, *p *<* *.001; Figure 5a,e,f). Specifically, we found a 67 ± 27% reduction after expression of the AAV‐ShRNATau (*p *<* *.0167). After blocking the expression of the AAV‐ShRNATau, the levels of TrkB phosphorylated at Tyr816 returned to baseline. Together, these data indicate that reducing tau levels leads to lower BDNF levels and reduced activation of TrkB receptors.

We also measured the levels of synaptophysin, another protein involved in memory formation and synaptic plasticity and found a significant difference among the three groups (*F*_(2,18)_ = 6.279, *p *<* *.05; Figure 5a,g). Specifically, we found a 41 ± 21% reduction in synaptophysin levels after expression of the AAV‐ShRNATau. After blocking the expression of the AAV‐ShRNATau, synaptophysin levels returned to baseline. We also found that PSD95 levels, a major scaffolding protein in the excitatory postsynaptic density (El‐Husseini, Schnell, Chetkovich, Nicoll & Bredt, 2000), were significantly different among the groups (*F*_(2,18)_ = 13.044, *p *<* *.001; Figure 5a,h). Specifically, there was a 46 ± 13% reduction in PSD95 levels after the expression of the AAV‐ShRNATau. We also found that after tau restoration, PSD95 levels were 70 ± 25% higher compared to control mice (*p *<* *.0167). These results suggest that knockdown of tau reduces the levels of critical proteins involved in memory formation and synaptic strength, which may explain deficits in learning and memory.

Previous work has shown that reduced dendritic spine density correlates with poor cognition (Mahmmoud et al., 2015). To examine the anatomical basis of cognitive deficits, we quantified synaptic connections by counting spines in the dendritic region of CA1 pyramidal neurons. To accomplish this, we processed brains using the Rapid Golgi kit, which impregnates neuronal cell bodies, axons, dendrites, and spines. We 3D‐reconstructed 48 pyramidal neurons (12 neurons per group, 3 animals/group), using Neurolucida software. All of the neurons resided in the dorsal part of the CA1 hippocampal region and were completely stained with Golgi along basal and apical dendrites. We found a significant effect of group for basal dendrite spine densities (*F*_(2,33)_ = 7.609, *p *<* *.05; Figure 5i‐j). We observed a 28 ± 10% reduction in basal dendritic spine densities after expression of the AAV‐ShRNATau (*p *<* *.0167). After blocking the expression of the AAV‐ShRNATau, basal dendritic spine levels returned to baseline. Similarly, we found a significant effect of group for apical dendrite spine densities (*F*_(2,33)_ = 4.475, *p *<* *.05; Figure 5i,k). We found a 24 ± 14% reduction in apical dendritic spine densities after expression of the AAV‐ShRNATau (*p *<* *.0167) and blocking the expression of the AAV‐ShRNATau retuned spine density to baseline. These results suggest that acute knockdown of tau may induce deficits in spatial learning and memory by reducing dendritic spine densities.

## 3 DISCUSSION

Understanding the consequence of tau knockdown at adulthood is critically important given the long‐standing debate of whether tau‐mediated behavioral deficits are due to toxic gain‐of‐function or to tau loss‐of function (Trojanowski & Lee, 2005). The data presented here unambiguously show, for the first time, that reducing tau levels in the hippocampus of adult mice impairs both motor coordination and spatial learning and memory. Our approach circumvents the developmental compensation issues observed in tau knockout models (Ke et al., 2012). Additionally, these findings suggest that cognitive and noncognitive deficits observed in AD and related tauopathies may be related to tau loss‐of‐function.

pro‐BDNF is cleaved to form the mature 14‐kDa BDNF form (Kowiański et al., 2017). However, changes in pro‐BDNF levels are not always directly linked to changes in BDNF levels. Further, pro‐BDNF and BDNF may have opposite effects on learning and memory. For example, a recent report showed that increasing pro‐BDNF levels leads to cognitive deficits (Buhusi, Etheredge, Granholm & Buhusi, 2017). In contrast, reduced BDNF levels also are associated with cognitive deficits (Kowiański et al., 2017). Here, we found that both BDNF and pro‐BDNF levels were reduced following tau knockdown and went back to baseline after we turned off the expression of the ShRNATau. Consistent with our results, stimulation of cultured rat hippocampal neurons with BDNF increases tau protein expression and promotes spine growth, while knocking down tau prevented the BDNF‐mediated spine growth (Chen et al., 2012).

Tau is also found at postsynaptic sites where it directly interacts with the PSD95/NMDA receptor complex (El‐Husseini et al., 2000; Hu et al., 2011). In primary hippocampal neurons, NMDA receptor activation leads to tau phosphorylation, which in turn interacts with PSD95 to promote synaptic plasticity (Hu et al., 2011). Notably, BDNF upregulation increases the size of PSD95 puncta in spines and the overall amount of PSD95 and synaptophysin (Hu et al., 2011; Yoshii & Constantine‐Paton, 2007). Thus, it is tempting to speculate that the decrease in tau levels reduces BDNF levels, which in turn reduces the amount of PSD95. Taken together, these results suggest that gain beyond baseline levels of BDNF and PSD95 may serve to increase the synaptic strength of neurons, thereby contributing to the rescue of cognitive deficits. In contrast, reduced BDNF levels in APP23 mice were rescued when these mice were crossed with tau knockout mice (Rosa et al., 2016). In contrast, while mice expressing mutant APP show reductions in PSD95 and synaptophysin levels (Oakley et al., 2006), it remains to be established whether knocking down tau rescues these changes.

Our results show that tau knockdown reduces dendritic spine density of CA1 pyramidal neurons, which likely contribute to the deficits in learning and memory. As discussed above, there is clear evidence for a physiological role of tau in synaptic plasticity (Frandemiche et al., 2014; Hu et al., 2011). Conversely, hyperphosphorylated tau accumulates in synaptic spines and alters synaptic function in a mouse model of AD (Hoover et al., 2010). Consistent with this observation, tau translocates to synaptic spines in an activity‐dependent manner, an event blocked by Aβ (Frandemiche et al., 2014). Taken together, these data clearly indicate that tau can contribute to cognitive deficits both by toxic gain‐of‐function and by loss‐of‐function.

While our work provides evidence that tau knockdown impairs learning and memory in wild‐type mice, a vast number of studies have shown that knockout of endogenous tau in AD mouse models protects against Aβ‐induced cognitive deficits (Ittner et al., 2010; Ke et al., 2012; Roberson et al., 2011; Vossel et al., 2015). For example, crossing APP mice with tau knockout mice improved spatial reference memory (Roberson et al., 2011). Mechanistically, these changes were linked to a reduced susceptibility to excitotoxicity in APP mice lacking the tau gene. In contrast, others have shown that loss of tau in APP mice causes neurodegeneration, motor, and memory deficits (Dawson et al., 2010). These results are in line with our finding and indicate an important role of tau in normal neuronal function.

A complex role of tau in learning and memory is also evident in tau transgenic mice. For example, mice expressing the entire human tau gene did not develop any frank neuropathology (Duff et al., 2000). However, removing endogenous mouse tau from these transgenic mice led to spatial memory deficits, perturbed LTP, as well as significant neuronal loss, ventricle enlargement, and reduced cortical thickness (Ando et al., 2011; Andorfer et al., 2005; Polydoro, Acker, Duff, Castillo & Davies, 2009). Strong evidence shows that transgenic mice overexpressing mutant tau develop spatial reference memory and motor impairments. These behavioral changes are associated with deficits in spine densities and mitochondrial dysfunction (Denk & Wade‐Martins, 2009; Kandimalla, Manczak, Yin, Wang & Reddy, 2018; Trojanowski & Lee, 2005). While these studies clearly show that overexpression of mutant tau is sufficient to cause learning and memory deficits, they do not address a possible interaction between the human transgene and the endogenous mouse tau.

The data presented here provide compelling evidence that tau is necessary for motor and cognitive function in the adult brain. These results suggest that developmental compensation of microtubule‐associated proteins in tau knockout models may mask the true behavioral phenotype of tau removal at adulthood. Additionally, these results firmly support that tau loss‐of‐function may contribute to the clinical manifestations observed in AD and related tauopathies. Given that antitau therapies are in clinical trials (ClinicalTrials.gov Identifier: NCT03186989; NCT03008161) to halt the production of NFTs, attention should be given as to whether they may simultaneously compromise healthy tau. These results may guide the development of future therapies for Alzheimer's disease and tauopathies to halt pathological tau while preserving healthy tau, thereby not further impairing motor and cognitive function in individuals suffering from these insidious diseases.

## 4 MATERIAL AND METHODS

### 4.1 Viral constructs generation

Adeno‐associated virus (AAV) vectors were generated by Vector BioLabs with the following constructs: AAV1‐CamKII‐rtTA‐Tet/U6‐Tau‐ShRNA and AAV1‐CamKII‐rtTA‐Tet/U6‐Scrm‐ShRNA. Overall, the Tet/U6 included seven Tet operator sequences. Notably, the ShRNA sequence for tau was obtained from a previous publication (Vossel et al., 2015). The experimental virus was generated to target the MAPT gene through the usage of a doxycycline‐inducible ShRNA. The final viral titer was 3.8 × 10^13^ GC/ml. The sequence of the AAV‐ShRNATau is G‐ACAGAGTCCAGTCGAAGATT‐CTCGAG‐AATCTTCGACTGGACTCTGTC‐TTTTTT. The sequence of the AAV‐ShRNAScrm is CACC‐GAACAAGATGAAGAGCACCAA‐CTCGAG‐TTGGTGCTCTTCATCTTGTTC‐TTTTTT.

### 4.2 Mice

We used 7‐month‐old C57Bl6/SJL (F1) female mice (Stock No. 100012, Jackson Laboratory) that were group‐housed 5 mice/cage. Male mice have a tendency to fight when grouped housed after surgery. To avoid fighting and injury after surgery, only female mice were used in this study. The mice were housed in the Arizona State University Animal Resource Center Facility. This facility operates in compliance with the USDA Animal Welfare Act, the Guide for the Care and Use of Laboratory Animals, under OLAW accreditation and IACUC approved protocols. All mice were housed up to five per cage at 23°C, kept on a 12‐h light/dark cycle and were given *ad libitum* access to food and water. We randomly assigned mice to a specific group, and there were no factors that determined the group selection. No mice were excluded from the statistical analyses. All procedures were approved by the Institutional Animal Care and Use Committee of the Arizona State University.

### 4.3 Stereotaxic surgeries

Mice were anesthetized with isoflurane and placed in a stereotaxic frame. Using a 5‐μl Hamilton syringe, we injected the AVVs using the following coordinates from bregma: −2.1 mm anteroposterior; ±1.8 mm lateral/medial; −1.5 mm dorsoventral from the skull. The AAVs (2 μl/hemisphere) were injected at 0.5 μl/min, after which the needle was left in place for three additional minutes before it was slowly removed.

### 4.4 Behavioral testing

*Open field* The open‐field test was conducted in a clear Plexiglas box (40 × 60 cm) to assess general motor function and anxiety as previously described (Caccamo, Maldonado, Bokov, Majumder & Oddo, 2010). *Rotarod* The rotarod test was conducted as previously described (Caccamo et al., 2010). Briefly, each mouse was trained for two consecutive days (six trials/day), followed by a probe trial. On the first and second day the rod accelerates from 0 to 15 rpm in 20 s and then maintains a speed of 15 rpm for up to 90 s. On the third day, the rod accelerates at 1 rpm/s for 90 s until the mouse falls off the rod. *Morris water maze* Mice were tested in a circular tank of 1.5 m in diameter located in a room with extra‐maze cues as detailed in Caccamo et al. (2010). During the rescue experiments, 10 mice/group were retested in the MWM task. The location of the escape platform was switched to a different quadrant, and extra‐maze cues were moved randomly to avoid any retest effects (Vorhees & Williams, 2006).

### 4.5 Protein extraction and western blots

Mouse proteins were prepared as previously described (Velazquez, Shaw, Caccamo & Oddo, 2016; Velazquez et al., 2017). One hemisphere of the brain was postfixed in 4% paraformaldehyde for 48 hr while the other hemisphere had the hippocampus and cortex isolated, flash‐frozen in dry ice, and stored at −80°C. A subset of hemispheres were dropped in Golgi–Cox solution following the manufacturer protocol (Rapid Golgi; FD NeuroTechnologies). The frozen brain regions were homogenized in ice‐cold T‐PER protein extraction buffer (Thermo Fisher Scientific) containing complete protease inhibitor (Roche Applied Science) and phosphatase inhibitor (Life Technologies). The homogenized mixtures were centrifuged at 100,000 *g* for 1 hr at 4°C. The resulting supernatant was recovered and stored at −80°C and used for Western blots, which were performed under reducing conditions using precast Novex gels (Life Technologies). Quantitative analyses of the Western blots were obtained by normalizing the intensity of the protein of interest with its loading control, β‐actin.

### 4.6 Brain tissue processing and histology

The immunohistochemistry was performed as we previously described (Velazquez et al., 2016). Hemispheres were fixed in 4% paraformaldehyde for 48 hr. Tissue was sectioned (50 μm thick) using a sliding vibratome and stored in 0.02% sodium azide in PBS. The endogenous peroxidase activity was quenched with 3% H_2_O_2_ in 10% methanol for 30 min. Tissue was incubated overnight at 4°C with an appropriate primary antibody. Sections were washed and incubated in the appropriate secondary antibody for 1 hr at room temperature. Sections were washed and incubated with diaminobenzidine substrate using the avidin–biotin horseradish peroxidase system (Vector Labs). The experimenter was blinded to the group allocation.

### 4.7 Golgi staining and dendritic spine quantification

To visualize dendrites and spines of pyramidal neurons, one hemisphere per animal was processed using the commercially available Golgi–Cox kit as described by the manufacturer (Rapid Golgi; FD NeuroTechnologies). Coronal sections (240 μm) were obtained using a freezing–sliding microtome, mounted on 2% gelatin‐coated glass slides, and stored in the dark at room temperature. After 2 days of drying, sections were rinsed, dehydrated, cleared with xylene, and coverslipped. Pyramidal neurons impregnated with the Golgi solution were readily identified in the dorsal hippocampal region by their characteristic triangular soma shape and numerous dendritic spines. CA1 neurons (three brains per group, four neurons per brain) were three‐dimensionally reconstructed by NeuroLucida Version 2017 Software (MicroBrightField). We quantified at least three basal and three apical dendrites per neuron. We used a 100× oil‐immersion objective to identify spines in dendrites longer than 200 μm. We calculated spine densities as mean numbers of spines per micrometer.

### 4.8 Antibodies

All antibodies used in these experiments were validated in mouse and/or human tissue by the manufacturers: from Clontech: TetR (catalog number 631132, dilution 1:1,000); from Cell Signaling: β‐actin (catalog number 4967, dilution 1:10,000), eIF2α (catalog number 2103, dilution 1:500), Phospho‐eIF2α Ser51 (catalog number 9721S, dilution 1:500), GFAP (catalog number 3670, dilution 1:200), PSD95 (catalog number 2507, dilution 1:1,000), and Tau46 (catalog number 4019, dilution 1:100); from Calbiochem: Tau5 (catalog number 577801, dilution 1:5,000); from Abcam: pro‐BDNF and mature BDNF (catalog number ab6201, dilution 1:1,000); from EMD Millipore: TrkB (catalog number 07‐225, dilution 1:500), phosphorylated TrkB at Tyr 816 (catalog number ABN1381, dilution 1:500), NeuN (catalog number MAB377, dilution 1:200), Synaptophysin (catalog number MAB5258, dilution 1:1,000); from Thermo Fisher: DAPI (catalog number D1306); and from Sigma‐Aldrich: MAP1A (catalog number M4278, dilution 1:500).

### 4.9 Statistical analyses

The performance data for the open‐field and rotarod task were analyzed using a Student's unpaired *t* test. The Morris water maze data were analyzed using a repeated‐measures ANOVA, followed by Bonferroni's corrected post hoc tests, when appropriate. The Western blot, immunostained, dendritic spine densities and biochemical data were analyzed using a one‐way ANOVA. These analyses were performed using Stat view for Windows version 5.0.1. Examination of descriptive statistics revealed no violation of any test assumptions that would warrant using statistical tests other than the ones used here. Significance was set at *p *<* *.05.

## CONFLICT OF INTEREST

The authors have no conflict of interest.
